# Thyroid-Stimulating Hormone, Age, and Tumor Size are Risk Factors for Progression During Active Surveillance of Low-Risk Papillary Thyroid Microcarcinoma in Adults

**DOI:** 10.1007/s00268-022-06770-z

**Published:** 2022-10-02

**Authors:** Yasuhiro Ito, Akira Miyauchi, Makoto Fujishima, Takuya Noda, Tsutomu Sano, Takahiro Sasaki, Taketoshi Kishi, Tomohiko Nakamura

**Affiliations:** 1 Department of Surgery, Kuma Hospital, 8-2-35, Shimoyamate-dori, Kobe, Hyogo 650-0011 Japan; 2 Department of Head and Neck Surgery, Kuma Hospital, Kobe, Hyogo 650-0011 Japan; 3 Department of Internal Medicine, Kuma Hospital, Kobe, Hyogo 650-0011 Japan; 4 Present Address: Department of Head and Neck Surgery, Kanazawa Medical University, Uchinada, Ishikawa 920-0293 Japan; 5 Present Address: Department of Otorhinolaryngology, Hyogo Prefectural Nishinomiya Hospital, Nishinomiya, Hyogo 662-0918 Japan; 6 Present Address: Department of Internal Medicine, Shiroyama Hospital, Habikino, Osaka 583-0872 Japan

## Abstract

**Background:**

Active surveillance (AS) of low-risk papillary thyroid microcarcinoma (PTMC) was initiated at Kuma Hospital in 1993 and is gradually spreading worldwide. We assessed the effect of thyroid-stimulating hormone (TSH) levels on PTMC enlargement in patients on AS.

**Methods:**

We enrolled 2705 patients with cytologically diagnosed PTMC who had undergone AS between January 2005 and July 2019. Patients with Graves disease were excluded. The median AS period was 5.5 years (range 1.0–15.7 years). Tumor enlargement was defined as a size increase ≥3 mm. Chi-square test, Kaplan–Meier method, log-rank test, Cox proportional hazard, and logistic regression were used to compare variables.

**Results:**

Ninety-two patients (3.4%) experienced tumor enlargement; the 5-, 10-, and 15-year enlargement rates were 3.0%, 5.5%, and 6.2%, respectively. Young age (<40 years, *p* < 0.001), large tumor size (≥9 mm, *p* = 0.017), and high detailed TSH score (≥3, higher than the lower normal limit, *p* = 0.011) were significant factors relating to tumor enlargement in the multivariate analysis. In a subset of patients aged <40 years, a low detailed TSH score (<3) was an independent factor against tumor enlargement (*p* = 0.039). Only 22 patients (0.8%) experienced novel lymph node metastasis; the 5-, 10-, and 15-year node metastasis rates were very low, at 0.9%, 1.1%, and 1.1%, respectively.

**Conclusions:**

Young patients with PTMC are more likely to experience tumor growth. Mild TSH suppression to achieve a low normal range may prevent carcinoma enlargement; however, prospective studies are needed to draw more reliable conclusions.

## Introduction

Papillary thyroid carcinoma (PTC) is the most common malignancy arising from the thyroid, and a PTC ≤ 10 mm is classified as papillary thyroid microcarcinoma (PTMC). Active surveillance (AS) of PTMC with no high-risk features (clinical node and/or distant metastasis and significant extrathyroid extension), namely low-risk PTMC (T1aN0M0), was initiated at Kuma Hospital (Kobe, Japan) in accordance with a proposal by Akira Miyauchi in 1993. In 1995, the Cancer Institute Hospital (Tokyo, Japan) also started AS, and favorable outcomes of patients under AS have been reported from the two hospitals [1–6]. With these promising results, the Japan Association of Endocrine Surgery and Japan Thyroid Association published consensus statements and a position paper supporting the implementation of AS for PTMC [7, 8]. Recently, AS has been adopted as a management strategy for PTMC in the guidelines constructed by the American Thyroid Association [9]; thereafter, several articles have been published on this issue from other countries [10–16].

Since most differentiated thyroid carcinomas retain a stimulatory response to circulating thyrotropin, thyroid-stimulating hormone (TSH) suppressive therapy has been widely adopted to prevent the growth of metastatic/recurring carcinoma lesions, especially for advanced differentiated thyroid carcinoma, including PTC [9, 17]. For the AS of PTMC, whether TSH levels affect carcinoma progression is an important clinical question. Two articles regarding this issue have been published; however, they presented discrepant conclusions [18, 19]. In this study, we aimed to evaluate the possible effect of TSH on PTMC progression using a detailed TSH score system modified from the original TSH score system reported by Cooper et al. [20].

## Materials and methods

### Patients

Between 1993 and 2019, 3312 patients with PTMC underwent AS at Kuma Hospital. In 2005, our hospital adopted an electronic medical record system. Subsequently, 2896 patients were diagnosed with PTMC by cytology (Bethesda V or VI) and underwent AS between January 2005 and July 2019. Patients who had comorbid Graves’ disease (*n* = 191) were excluded, and the remaining 2705 were enrolled (353 men and 2352 women; age: median, 58 years; range 20–92 years). The median AS period was 5.5 years (range 1.0–15.7 years). Patients aged <20 years and those who had undergone AS for <1.0 year were excluded. Patients who had undergone AS for suspected PTMC tumors determined by ultrasound examinations but lacking cytological diagnoses were excluded.

### Active surveillance

AS was performed on patients diagnosed with PTMC who opted for this management, as previously described [1]. Briefly, patients were instructed to visit our outpatient clinic once/twice per year to undergo blood and ultrasound examinations. Ultrasound was performed to determine any tumor size changes and to assess any new formations in suspicious lymph nodes. The average of the tumor sizes at the first and second examinations was considered the baseline to minimize observer variations. For the same reason, when a size increase ≥3 mm compared with the baseline was detected in two successive ultrasound examinations, the tumor was defined as enlarged at the point of the first ultrasound showing enlargement. Conversion surgery was discussed with patients exhibiting tumor enlargement. If a patient preferred to remain on AS, it was continued until the tumor reached 13 mm. When a suspicious lymph node was detected, cytological examination was performed and conversion surgery was recommended if the node was diagnosed as a PTC metastasis.

### Establishment of the detailed TSH score

TSH level was measured using an Architect TSH (Abbott, Japan LLC, Tokyo, Japan) until December 2018 and an Elecsys TSH (Roche Diagnostics KK, Tokyo, Japan) after January 2019, according to the manufacturers’ recommendations. These assays are well approved and used in many counties. However, there are differences between them, specifically, the lowest detection levels were <0.003 mIU/mL until 2018 (Architect) and <0.005 mIU/mL after 2019 (Elecsys), and the ranges of normal values were 0.3–5 mIU/mL until 2018 (Architect) and 0.5–5 mIU/mL after 2019 (Elecsys). Cooper et al. created a TSH score system for the evaluation of the possible effects of TSH on disease progression in differentiated thyroid cancer [20], which has been used by others [21]. To minimize the bias of the different measurement values, we adopted the TSH score instead of actual TSH values. Since we wanted to analyze the possible effect of TSH in detail, we modified the original score system into a novel detailed TSH score system. For the new system, TSH scores are defined as the following: (1) lower than the detection limit; (2) detectable and <0.05 mIU/mL; 2.5, ≥0.05 mIU/mL and less than the lower normal limit; (3) within the normal range and lower than the mean of the normal ranges; (3.5) within the normal range and higher than the mean of the normal ranges; and (4) higher than the upper normal limit. Since the intervals between patients’ visits were uneven, they were considered when the average TSH score was calculated. This was done using the following formula:

$$\sum\limits_{1}^{n} {\frac{{{\text{TS}}_{n} + {\text{TS}}_{n + 1} }}{2}} \times {\raise0.7ex\hbox{${{\text{ID}}_{n} }$} \!\mathord{\left/ {\vphantom {{{\text{ID}}_{n} } {\sum\nolimits_{1}^{n} {{\text{ID}}} }}}\right.\kern-\nulldelimiterspace} \!\lower0.7ex\hbox{${\sum\nolimits_{1}^{n} {{\text{ID}}} }$}}$$

where ID_*n*_ is each interval (days) and TS_*n*_ and TS_*n*+1_ are the TSH scores from the beginning and end of each interval, respectively. The mean TSH score for each interval was multiplied by interval days, and the sum of these values was divided by the total days of the study period for each patient. This value is defined as an interval-adjusted detailed TSH score.

### Statistical analysis

StatFlex software (Artec, Osaka, Japan) was used to perform univariate and multivariate analyses. The Chi-square test was used to compare variables. For time series and univariate analyses, the Kaplan–Meier method and log-rank test were used. Univariate Cox proportional hazard models were performed to identify independent prognostic factors. Multivariate logistic regression was then performed on factors with *p* values of <0.20. A *p* value < 0.05 was considered statistically significant.

## Results

### Demographic and clinical characteristics of the patients

Table 1 includes the demographic and clinical characteristics of the 2705 patients. No patient experienced distant recurrence or died because of thyroid carcinoma during AS. Levothyroxine was administered to 338 and 226 patients at the initiation of AS and during AS, respectively, mainly to improve hypothyroidism or mildly suppress TSH in order to prevent tumor enlargement.

**Table 1:** Demographics and clinical characteristics of enrolled patients

Variables	Number of patients (*n* = 2705)
*Sex*	
Male	353 (13.0)
Female	2352 (87.0)
*Age*	
<40 years	313 (11.6)
40–59 years	1132 (41.8)
≥60 years	1260 (46.6)
*Family history of PTC*^*a*^	
Yes	101 (3.7)
No	2604 (96.3)
*Multiplicity*^*b*^	
Yes	391 (14.5)
No	2314 (85.5)
*Chronic thyroiditis*^*c*^	
Yes	900 (33.3)
No	1805 (66.7)
*Levothyroxine administration at the beginning of AS*^*d*^	
Yes	338 (12.5)
No	2367 (87.5)
*Initiation of levothyroxine administration during AS*^*e*^	
Yes	226 (9.5)
No	2141 (90.5)
*Tumor size at diagnosis*	
<5 mm	385 (13.9)
5–8 mm	1849 (68.7)
9–10 mm	471 (17.4)

Values are presented as *n* (%)

*AS* active surveillance, *PTC* papillary thyroid carcinoma

^a^One or more first-degree relative had papillary thyroid carcinoma

^b^Evaluated by imaging techniques (mainly ultrasound)

^c^Positive for anti-thyroglobulin antibody and/or thyroid peroxidase antibody

^d^Administered before AS or within 6 months of AS initiation

^e^A total of 138 patients who were given levothyroxine at the beginning of AS were excluded from the calculation

### Tumor enlargement outcomes

**Fig. 1:**
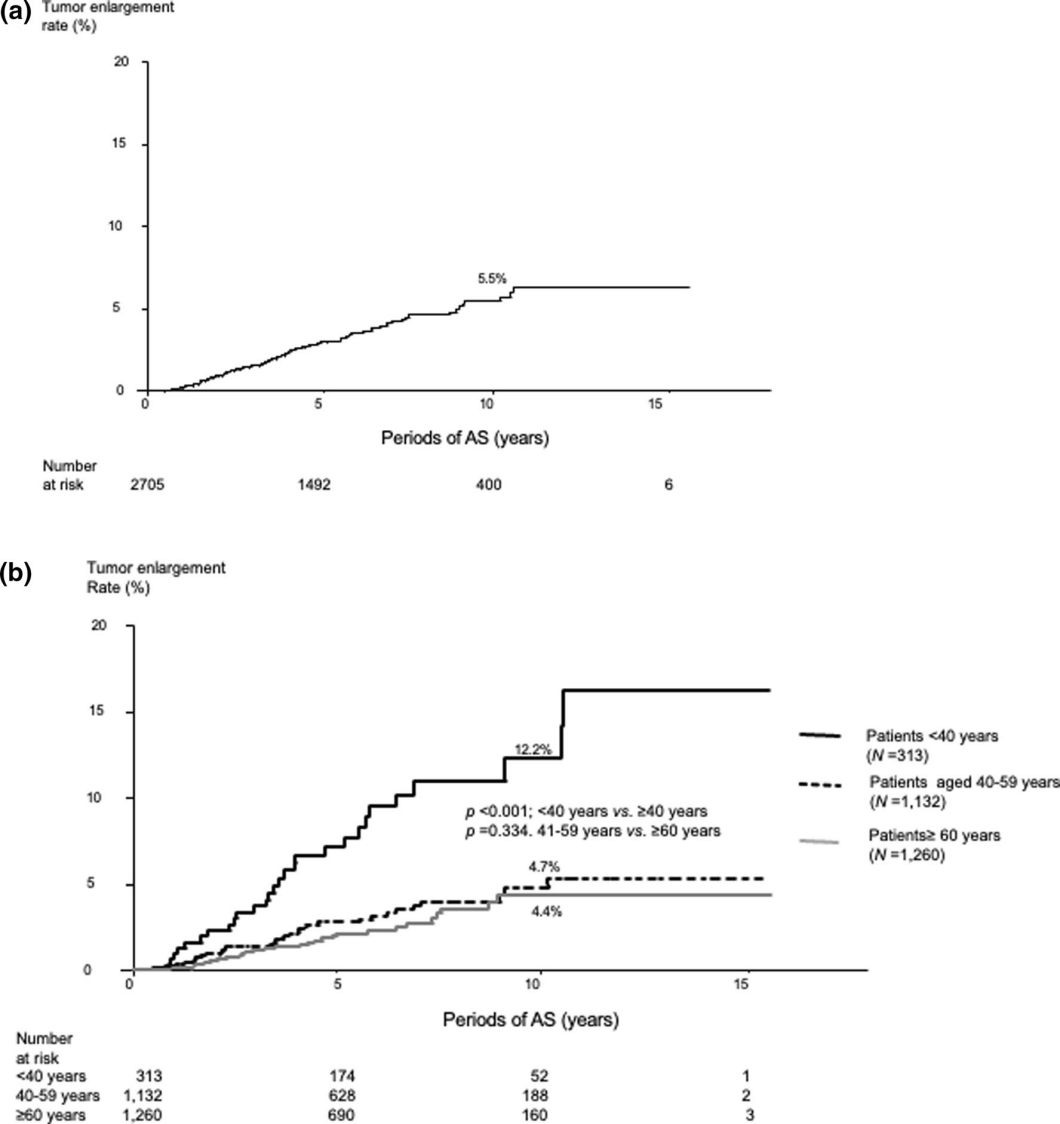
Kaplan–Meier curve of **a** overall tumor enlargement rates for 2705 PTMC patients and **b** tumor enlargement rates for PTMC patients according to patient age

Ninety-two patients (3.4%) experienced tumor enlargement. The 5-, 10-, and 15-year tumor enlargement rates were 3.0%, 5.5%, and 6.3%, respectively (Fig. 1a). Table 2 shows the 5-, 10-, and 15-year enlargement rates in patients aged <40, 40–59, and ≥60 years. The PTMCs in patients aged <40 years were more likely to enlarge than those in patients aged ≥40 years (*p* < 0.001); however, there was no significant difference in the enlargement rates between patients aged 40–59 years and those aged ≥60 years (*p* = 0.334) (Fig. 1b). Table 3 presents the univariate and multivariate analyses on the relationship between various clinicopathological features and tumor enlargement. The univariate analysis revealed that age <40 years, tumor size ≥9 mm, and a detailed TSH score ≥3 were significantly related to tumor enlargement. The multivariate analysis revealed that these three factors independently predicted tumor enlargement, with the highest hazard ratio for those aged <40 years, followed by a detailed TSH score ≥3. Table 4 shows a subset analysis of factors affecting tumor enlargement in patients aged <40 years. A TSH score ≥3 was an independent predictor of tumor enlargement (hazard ratio 4.574); in other words, PTMC in patients with a TSH score <3 was less likely to enlarge. We conducted the same analysis for patients aged ≥40 years. The multivariate analysis of the factors with *p *values <0.20 in the univariate analysis (multiplicity, *p* = 0.147; tumor size ≥9 mm, *p* = 0.100; TSH score ≥3, *p* = 0.156) revealed that none of the factors were significant independent predictors for tumor enlargement.

**Table 2:** Tumor enlargement rates of papillary thyroid microcarcinoma according to patient age

Periods of AS	Entire series	<40 years	40–59 years	≥60 years
5 years	3.0	7.1	2.7	2.1
10 years	5.5	12.2	4.7	4.4
15 years	6.3	16.2	5.2	4.4

Values are presented as %

*AS* active surveillance

**Table 3:** Univariate and multivariate analyses* for factors affecting papillary thyroid microcarcinoma tumor enlargement

Clinicopathological features	*p* values for univariate analysis	*p* values for multivariate analysis	Hazard ratio (95% CI)
Male sex	0.531		
**Age <40 years**	**<0.001**	**<0.001**	**3.704 (2.367–5.797)**
Chronic thyroiditis	0.993		
Family history of PTC	0.959		
Multiplicity	0.522		
Levothyroxine administration at the beginning of AS	0.410		
Tumor size <5 mm	0.863		
**Tumor size **≥**9 mm**	**0.029**	**0.017**	**1.790 (1.107–2.895)**
**Detailed TSH score **≥**3**	**0.047**	**0.011**	**2.954 (1.282–6.802)**

Bold indicates statistical significance

*AS* active surveillance, *CI* confidence interval, *PTC* papillary thyroid carcinoma, *TSH* thyroid-stimulating hormone

*Factors with *p* < 0.20 in the univariate analysis were adopted for the multivariate analysis

**Table 4:** Univariate and multivariate analyses* of factors affecting papillary thyroid microcarcinoma tumor enlargement in patients aged ≤ 40 years

Clinicopathological features	*p* values for univariate analysis	*p* values for multivariate analysis	Hazard ratio (95% CI)
Male sex	0.899		
Chronic thyroiditis	0.155	0.286	0.660 (0.307–1.416)
Family history of PTC	0.706		
Multiplicity	0.426		
Levothyroxine administration at the beginning of AS	0.856		
Tumor size <5 mm	0.916		
Tumor size ≥9 mm	0.080	0.068	2.223 (0.942–5.248)
**Detailed TSH score **≥**3**	**0.034**	**0.039**	**4.574 (1.076–19.439)**

Bold indicates statistical significance

*AS* active surveillance, *CI* confidence interval, *PTC* papillary thyroid carcinoma, *TSH* thyroid-stimulating hormone

*Factors with *p* < 0.20 in the univariate analysis were adopted for the multivariate analysis

### Patient outcomes in terms of lymph node metastasis

**Fig. 2:**
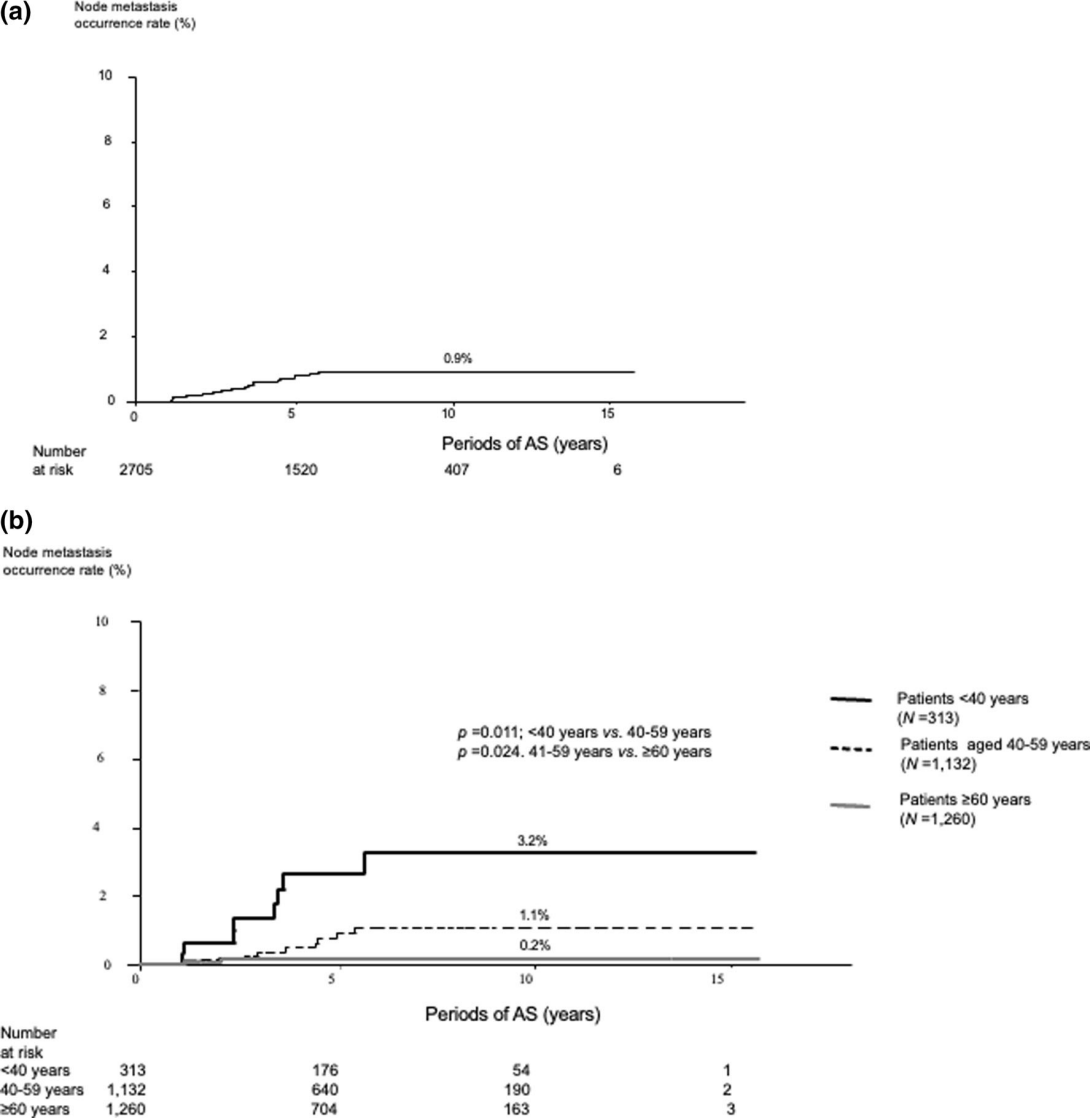
Kaplan–Meier curve of node metastasis occurrence rate for **a** 2705 PTMC patients and **b** PTMC patients according to patient age

Only 19 patients (0.7%) experienced a novel occurrence of lymph node metastasis. The 5-, 10-, and 15-year node metastasis appearance rates were 0.8%, 0.9%, and 0.9%, respectively (Fig. 2a). Table 5 shows the occurrence rates of patients aged <40, 40–59, and ≥60 years. The rates significantly decreased with age (*p* = 0.011, between patients <40 and 40–59 years, *p* = 0.024 between patients 40–59 and ≥60 years) (Fig. 2b). For the multivariate analysis, age <40 years and male sex were independent predictors of novel nodal metastasis (Table 6). Only multiplicity was an independent predictor of node metastasis occurrence for patients aged <40 years (Table 7). In patients aged ≥40 years, male sex and age of 40–59 years were significantly related to node metastasis for univariate analysis. For multivariate analysis, both were significant independent predictors of node metastasis (Table 8). Three patients experienced both tumor enlargement and metastasis: two experienced novel node metastasis after tumor enlargement and one experienced both simultaneously.

**Table 5:** Node metastasis occurrence rates of papillary thyroid microcarcinoma according to patient age

Periods of AS	Entire series	<40 years	40–59 years	≥60 years
5 years	0.8	2.6	0.9	0.2
10 years	0.9	3.2	1.1	0.2
15 years	0.9	3.2	1.1	0.2

Values are presented as %

*AS* active surveillance

**Table 6:** Univariate and multivariate analyses* for factors affecting novel nodal papillary thyroid microcarcinoma metastasis

Clinicopathological features	*p* values for univariate analysis	*p* values for multivariate analysis	Hazard ratio (95% CI)
**Male sex**	**0.016**	**0.005**	**4.089 (1.536–10.882)**
**Age <40 years**	**<0.001**	**<0.001**	**6.636 (2.634–16.719)**
Chronic thyroiditis	0.257		
Family history of PTC	0.990		
Multiplicity	0.187	0.100	2.368 (0.848–6.611)
Levothyroxine administration at the beginning of AS	0.374		
Tumor size < 5 mm	0.894		
Tumor size ≥9 mm	0.937		
Detailed TSH score ≥ 3	0.360		

Bold indicates statistical significance

*AS* active surveillance, *CI* confidence interval, *PTC* papillary thyroid carcinoma, *TSH* thyroid-stimulating hormone

*Factors with *p* < 0.20 in the univariate analysis were adopted for the multivariate analysis

**Table 7:** Univariate and multivariate analyses* for factors affecting novel nodal papillary thyroid microcarcinoma metastasis in patients aged <40 years

Clinicopathological features	*p* values for univariate analysis	*p* values for multivariate analysis	Hazard ratio (95% CI)
Male sex	0.078	0.097	3.988 (0.780–20.380)
Chronic thyroiditis	0.545		
Family history of PTC	0.990		
**Multiplicity**	**0.004**	**0.005**	**7.382 (1.814–30.050)**
Levothyroxine administration at the beginning of AS	0.990		
Tumor size <5 mm	0.116	0.236	2.412 (0.563–10.330)
Tumor size ≥9 mm	0.874		
Detailed TSH score ≥3	0.941		

Bold indicates statistical significance

*AS* active surveillance, *CI* confidence interval, *PTC* papillary thyroid carcinoma, *TSH* thyroid-stimulating hormone

*Factors with *p* < 0.20 in the univariate analysis were adopted for the multivariate analysis

**Table 8:** Univariate and multivariate analyses* for factors affecting novel nodal papillary thyroid microcarcinoma metastasis in patients aged ≥40 years

Clinicopathological features	*p* values for univariate analysis	*p* values for multivariate analysis	Hazard ratio (95% CI)
**Male sex**	**0.032**	**0.030**	**3.988 (0.780–20.380)**
Chronic thyroiditis	0.795		
Family history of PTC	0.990		
Multiplicity	0.549		
Levothyroxine administration at the beginning of AS	0.802		
Tumor size <5 mm	0.990		
Tumor size ≥9 mm	0.896		
Detailed TSH score ≥3	0.567		
**Age 40–59 years**	**0.041**	**0.040**	**4.990 (1.078–23.096)**

Bold indicates statistical significance

*AS* active surveillance, *CI* confidence interval, *PTC* papillary thyroid carcinoma, *TSH* thyroid-stimulating hormone

*Factors with *p* < 0.20 in the univariate analysis were adopted for the multivariate analysis

### Levothyroxine therapy and detailed TSH score

The TSH scores of the patients on levothyroxine were significantly lower than those not on it. In patients aged ≥40 years, the TSH scores of patients on levothyroxine were significantly lower (*p* < 0.0001) than that of those who were not on it. However, the TSH scores did not differ between the two groups in patients aged <40 years (Table 9).

**Table 9:** Relationship between levothyroxine therapy and detailed TSH scores in papillary thyroid microcarcinoma patients

Overall (*n* = 2705)			Age <40 years (*n* = 313)			Age ≥40 years (*n* = 2392)		
*Levothyroxine therapy*								
Yes (*n* = 338)	No (*n* = 2367)	*p* value	Yes (*n* = 62)	No (*n* = 251)	*p* value	Yes (*n* = 276)	No (*n* = 2.116)	*p* value
*Detailed TSH score*								
2.980 (0.245)	3.112 (0.227)	<0.001	3.038 (0.210)	3.039 (0.213)	0.995	2.966 (0.250)	3.121 (0.227)	<0.001

Values are presented as mean (standard deviation)

*TSH* thyroid-stimulating hormone

### Prognosis of patients who underwent conversion surgery

To date, 242 patients underwent conversion surgery after AS for ≥1 year. Conversion surgery was recommended to 72 and 170 patients because of disease progression and various other reasons, respectively. The periods between the beginning of AS and surgery ranged from 1.0 to 15.5 (median 5.1) years. Only one patient had lymph node recurrence after surgery (postoperative follow-up period, 0.1–13.8 years; median, 4.6 years), which was successfully treated with salvage surgery.

## Discussion

We analyzed the prognostic factors of tumor enlargement and node metastasis in 2705 PTMC patients. Our results showed that 5-, 10-, and 15-year tumor enlargement rates were 3.0%, 5.5%, and 6.3%, respectively. These incidences were considerably lower than those in our earlier study, which had a 10-year enlargement rate of 8.0% [1]. This could be due to the higher number of enrolled patients in this study. Further, we regarded a tumor as enlarged when a size increase ≥3 mm was detected in two successive examinations to minimize observer variations. This may also be a reason for the difference in enlargement rates between these studies. The 5-, 10-, and 15-year node metastasis rates were 0.8%, 0.9%, and 0.9%, respectively, which was also much lower than our earlier study, which had a 10-year node metastasis rate of 3.8% [1]. Node metastasis was defined only when a suspicious node was cytologically diagnosed as PTC. Whether cytological examinations were performed for suspicious nodes was completely based on the physicians’ discretion. In the past, physicians might have been cautious of node metastasis, and cytological examination would have been performed even for tiny suspicious nodes. Currently, physicians may only order a fine-needle aspiration for a suspicious node if it is relatively large. With the accumulation of experience with AS at the Kuma Hospital, physicians have come to understand that tiny suspicious nodes often remain small and do not cause any clinical problems. Patients tend to continue AS unless the node or multiple nodes begin to enlarge. Currently, fine-needle aspirations are typically ordered for suspicious nodes measuring ≥5 mm in the smallest dimension of the three dimensions of ultrasound examination.

Young age was a significant predictor of PTMC progression, which is consistent with previous reports [1, 11, 22]. In this study, we performed analyses not only for the entire study population, but also for patient age subsets. To evaluate TSH levels, we created a detailed TSH score system and evaluated it with interval adjustment. A high detailed TSH score was independently associated with tumor enlargement not only for the entire study population but also in the subset of patients aged <40 years. In other words, appropriate TSH suppression to low normal or mild subnormal levels may be effective in preventing tumor enlargement, especially for young patients. Based on these findings, although no prospective studies have been conducted, levothyroxine therapy for mild TSH suppression in young patients with high TSH levels may be effective in preventing PTMC enlargement. However, in contrast to patients aged ≥40 years, the mean detailed TSH scores of patients aged <40 years who were taking levothyroxine did not significantly differ from that of those who were not taking it (Table 9). Slightly increasing the dose of levothyroxine may be recommended in young patients with PTMC in order to achieve low normal or subnormal TSH levels.

In contrast to tumor enlargement, the detailed TSH score was not related to node metastasis. Miyauchi et al. showed that tumor size increase and node metastasis did not necessarily correlate [23]. Additionally, in the present study, only three PTMCs exhibited both tumor enlargement and node metastasis. Although conversion surgery is not always performed for PTMC tumors that enlarge ≥3 mm, we generally recommend immediate surgery for PTMC with novel occurrences of node metastasis that are confirmed by cytological examination according to physicians’ discretion and are diagnosed as metastasis. Thus, whether tumors undergoing node metastasis would also enlarge thereafter is unknown. The question remains whether tumor enlargement and node metastasis are related to each other. However, according to the data presented by Miyauchi et al. [23] and the present study, these events do not necessarily occur in parallel.

Previous studies reported discrepant results regarding the relationship between TSH levels and PTMC enlargement [18, 19]. Sugitani et al. demonstrated that baseline and mean TSH levels were not related to PTMC progression [18]. Kim et al. established a time-weighted average of serum TSH (TW-TSH) and showed that the highest (third tertile) TW-TSH was significantly related to PTMC progression (size increase ≥3 mm or novel appearance of node metastasis) for both univariate and multivariate analyses [19]. However, the number of enrolled patients was small (*n* = 127) and the median follow-up period was short (25 months). Further, they analyzed the size increase and node metastasis as a single group, possibly because of the small number of progression events.

According to the Cancer Incidence of Japan study conducted by the Ministry of Health, Labour and Welfare, the incidence rate of thyroid carcinoma per 10,000 population was 5.6% for male and 16.7% for female in 2018 [24]. In our patient series, the incidence was much higher than that in females (87.0%), similar to our previous publications [1, 25]. The reason for the discrepancy remains unclear, but females have more chance to undergo thyroid ultrasound together with the breast at a checkup for breast carcinoma. This may be one of the reasons for this phenomenon.

Our study had some limitations. Although AS was performed prospectively, administration of levothyroxine was decided at the physicians’ discretion and the analysis of TSH levels was retrospective. At the initiation of AS, we did not set optimal TSH values for each patient. Since we only enrolled PTMC patients who had undergone AS after the introduction of the electronic medical record system in 2005, the periods for patients who had undergone AS were comparably short (median 5.5 years). However, by using electronic medical records, we were able to analyze a large amount of data from a large number of patients in detail. With this advantage, we were able to determine that mild TSH suppression was effective for preventing the enlargement of PTMC, especially for young patients.

PTMC in young patients (aged <40 years) was more likely to progress than that in middle-aged and old patients. Especially for young patients, achieving low normal or mild subnormal TSH levels by levothyroxine therapy may be considered for suppression of tumor enlargement. For middle-aged and older patients, avoiding high TSH levels may be a sensible choice. Further prospective studies are necessary to strengthen our findings.
