# Correction: Thyroid-Stimulating Hormone, Age, and Tumor Size are Risk Factors for Progression During Active Surveillance of Low-Risk Papillary Thyroid Microcarcinoma in Adults

**DOI:** 10.1007/s00268-022-06861-x

**Published:** 2022-12-13

**Authors:** Yasuhiro Ito, Akira Miyauchi, Makoto Fujishima, Takuya Noda, Tsutomu Sano, Takahiro Sasaki, Taketoshi Kishi, Tomohiko Nakamura

**Affiliations:** 1 Department of Surgery, Kuma Hospital, 8-2-35, Shimoyamate-Dori, Kobe, Hyogo 650-0011 Japan; 2 Department of Head and Neck Surgery, Kuma Hospital, Kobe, Hyogo 650-0011 Japan; 3 Department of Internal Medicine, Kuma Hospital, Kobe, Hyogo 650-0011 Japan; 4 Present Address: Department of Head and Neck Surgery, Kanazawa Medical University, Uchinada, Ishikawa, 920-0293 Japan; 5 Present Address: Department of Otorhinolaryngology, Hyogo Prefectural Nishinomiya Hospital, Nishinomiya, Hyogo 662-0918 Japan; 6 Present Address: Department of Internal Medicine, Shiroyama Hospital, Habikino, Osaka 583-0872 Japan

**Correction to: World J Surg** 10.1007/s00268-022-06770-z

In the original online version of this article the values of family history of PTC in Table 1 were incorrect.

The original article was corrected.

